# Effect and influencing factors of early ileostomy closure 2–4 weeks on patients with rectal cancer

**DOI:** 10.3389/fonc.2026.1755035

**Published:** 2026-06-03

**Authors:** Shengze Li, Junjie An, Jin Tang, Zimei Hou, Bo Shi, Jiming Duan, Bianbian Qiao, Wenxing Li

**Affiliations:** 1 Department of General Surgery, The Second Hospital of Shanxi Medical University, Taiyuan, China; 2 The Second Clinical Medical College of Shanxi Medical University, Taiyuan, China; 3 Gastrointestinal, Pancreatic, Hernia & Abdominal Wall Surgery, The Fifth Clinical College of Shanxi Medical University, Taiyuan, China; 4 Department of Colorectal Anal Surgery, The Fifth Clinical Medical College of Shanxi Medical University, Taiyuan, China; 5 Department of Colorectal Anal Surgery, Taiyuan Seventh People’s Hospital, Taiyuan, China; 6 Shanxi Key Laboratory of Molecular Diagnosis and Therapy for Hematologic Diseases, Taiyuan, China

**Keywords:** complications, early closure, ileostomy, prediction model, rectal neoplasms

## Abstract

**Background:**

Early ileostomy closure(EIC) should be performed in selected patients. However, the optimal timing for EIC and which specific patients could benefit from it remains controversial. The aim of the study was to explore the safety and effect of 2–4 weeks ileostomy closure and influencing factors.

**Methods:**

This multicenter retrospective study conducted at three hospitals in China included 428 patients with underwent laparoscopic radical resection for rectal cancer followed by protective loop ileostomy. According to the time of ileostomy closure, the patients were divided into early closure (EC;2–4 weeks after operation) and routine closure(RC; 3–6 months after operation). A multivariate logistic regression model was used to analyze independent risk factors for EIC. A one-to-one propensity score matching (PSM) analysis was performed to balance baseline differences between groups. Primary endpoint was post-ileostomy closure complications. Secondary endpoints were stoma-related complications, perioperative outcomes, anal function and quality of life with the 36-item Short Form Health Survey(SF-36).

**Results:**

A total of 428 patients were included, with 94 receiving EC and 334 receiving RC. Multivariate logistic regression analysis screened out age, tumor site, neoadjuvant chemoradiotherapy, and pathological tumor-node-metastasis (pTNM) stage as independent influencing factors for EIC. The Nomogram was established based on these four factors. The area under the curve (AUC) of the model was 0.756 (95%CI: 0.70–0.811). The decision curve analysis (DCA) also showed good clinical practicality. After PSM, 89 pairs were matched. There was no significant difference between two groups regarding the post-ileostomy closure complications and anal function. EC group had significantly lower stoma-related complications(*P* < 0.05), shorter operative time (*P* < 0.05) and higher SF-36 quality of life score (*P* < 0.001) compared to RC group.

**Conclusion:**

Early closure of ileostomy at 2–4 weeks is safe and feasible in selected patients. The Nomogram prediction model that incorporates age, tumor site, neoadjuvant chemoradiotherapy and pTNM stage can effectively predict EIC in rectal cancer, and can perform effective preoperative stratification to guide clinical decision-making and personalized treatment.

## 1 Introduction

Colorectal cancer(CRC) represents the third most common cancer about 60% are diagnosed with rectal cancer (1). The global prevalence of CRC diagnosed in younger individuals is projected to increase (2).Anterior resection (AR) is standard procedure for upper rectal cancer, while low anterior resection (LAR) is the standard procedure for distal rectal cancer allowing anal sphincter preservation (3). Anastomotic leakage (AL) is a severe complication after CRC surgery and defined as a defect of the colorectal anastomosis confirmed by imaging, endoscopy, or re-operation, with an incidence of 3–24% (4, 5). Preventive Ileostomy, the most common procedure to reduce the rates of reoperation for AL, is associated with the risk of permanent stoma and an additional operation for stoma reversal (6). It not only brings complications related to the stoma, but also has an impact on the patient’s quality of life and mental health (7). The best timing for closure remains unclear. Previous studies reported routine closure (RC; 3–6 months after ileostomy) is recommended to perform (8). However, it fails to take into account the differences in treatment methods and the heterogeneity of patients. Current indications for neoadjuvant chemoradiotherapy (NACRT) in rectal cancer remains the standard for locally advanced rectal cancer (cT3–4 and/or N+) and have evolved to encompass a more selective approach based on high-resolution magnetic resonance imaging(MRI) staging (9).The use of MRI as the gold standard for staging now allows for better selection of patients with favorable prognosis who may proceed directly to surgery without neoadjuvant therapy (10). Preoperative high-resolution MRI staging has driven an evolution in patient selection, with patients who have favorable prognostic features are increasingly considered for early stoma closure. Multiple systematic reviews and meta-analyses support the safety and feasibility of early closure in precisely selected patients (11–13). Patient selection requires good perioperative counselling and shared decision-making (14).

In recent years, the strategy of Early ileostomy closure (EIC) is proposed (15). It benefits from the application of enhanced recovery after surgery(ERAS) program (16) and the continuous progress of novel surgical techniques (17). Multiple studies have demonstrated the safety and feasibility of EIC (15, 18). A recent systematic review and meta-analysis demonstrated comparable safety and functional outcomes between early and late closure after rectal cancer surgery, with no increased morbidity risk (19). Current research mainly focuses on 2 weeks and 1 month(4 weeks). Some studies supported that colorectal anastomosis, which is confirmed to be intact endoscopically and radiologically, can be safely closed 2 weeks postoperatively (20). Danielsen AK et al. (21)conducted a multicenter randomized controlled trial to compared early closure (8–13 days) with standard procedure (closure after > 12 weeks) after rectal resection for cancer. Their results indicated that it is safe to close a temporary ileostomy 8–13 days in selected patients without clinical or radiological signs of anastomotic leakage. However, some studies suggested that EIC 2 weeks appears to be associated with an increased postoperative incision infection (22), a higher risk of colorectal anastomotic leakage (23). The idea of two operations in two weeks being too taxing on the body was deemed the biggest disadvantage (24). EIC(4 weeks) is feasible (25) but the overall postoperative morbidity rate was dramatically higher than routine closure (8).

Little is known about the effect of 2–4 weeks ileostomy closure. Some studies have suggested that patients could benefit from early closure before starting the adjuvant chemotherapy in regard to long term oncological survival due to a higher rate of completeness of adjuvant chemotherapy treatment and thus better effectiveness (26). Compared to the 2 weeks, 2–4 weeks closure avoids the period when short-term complications such AL tend to occur in clusters, and is more acceptable to the patients. When early closure was performed within 2 weeks but not within 3 to 4 weeks EC had higher odds of colorectal anastomotic leakage than RC (23). Meanwhile, it is before starting the adjuvant chemotherapy and does not affect the implementation of the adjuvant chemotherapy compared to the 4 weeks closure or later. Previous research results have shown that improved surgeon technical skills may result in better stoma patient outcomes (27). We have implemented a series of measures that have been proven effective to reduce the incidence of AL, including preservation of left colic artery (28), transanal drainage tubes (29), anastomosis with tension-free and good blood supply (30), leak test intraoperatively (31).Some studies have demonstrated that the one-stitch method of protective loop ileostomy reduce the incidence rate of postoperative incision infection (32) and the surgical difficulty of returning the stoma in body mass index(BMI) obesity patients (33). The modified one-stitch method of protective loop ileostomy primarily enhances the support and positioning of the stoma, which has been shown in some studies to significantly reduces stoma dermatitis and prolapse rates (34, 35). In our study, we implemented the modified approach.

In this study, we have proposed a new strategy that EIC is performed 2–4 weeks in selected patients. We conducted a retrospective analysis of EIC at 2–4 weeks in multicenter. Therefore, this study aimed to compare the effects and safety of early closure (2–4 weeks) of the stoma in patients with rectal cancer and influencing factors.

## 2 Patients and methods

### 2.1 Study setting and period

This multicenter, retrospective study was conducted at 3 institutions in China, including The Second Hospital of Shanxi Medical University, The Fifth Clinical Medical College of Shanxi Medical University and Taiyuan Seventh People’s Hospital. All surgeons at three institutions underwent standardized training in rectal cancer surgery based on identical guidelines, ensuring procedural consistency. The data of patients who underwent preventive ileostomy reversal surgery for rectal cancer were collected between January 2019 and October 2024.

### 2.2 Eligibility criteria

#### 2.2.1 Inclusion criteria

(i)Diagnosed with rectal adenocarcinoma by the pathological diagnosis. (ii) Underwent laparoscopic radical resection of rectal cancer and performed prophylactic ileostomy. (iii) Before the operation, all patients were informed of the condition of the anastomosis through preoperative examination. (iv) with no severe liver or kidney function impairment, abnormal coagulation function, or nutritional risk.

#### 2.2.2 Exclusion criteria

(i) Patients who are unable to tolerate the surgery due to severe heart, lung or kidney disorders. (ii) With local recurrence or distant metastasis of the tumor during the preoperative examination. (iii) Preventive stoma after rectal repair surgery due to injuries or other causes. (iv) Unplanned reoperation with ileostomy.

### 2.3 Study population

Based on inclusion and exclusion criteria, a total of 428 eligible participants were included in the final analysis. We included 428 patients who were divided into two groups based on the time of ileostomy reversal: those with EC(2 weeks<time<4 weeks) and those with RC(3 months<time<6 months). To minimize selection bias when comparing outcomes between EC and RC in rectal cancer patients, we conducted a one-to-one propensity score matching (PSM) analysis to balance baseline characteristics.

### 2.4 Variables

The following information was retrieved from electronic records relating to patient demographics, pre-morbidities, use of systemic therapy, stoma characteristics and post-operative complications. Anastomotic leakage was defined according to the International Study Group of Rectal Cancer (ISREC) classification, encompassing grades A to C, and was diagnosed within the following follow-up intervals: patients were assessed weekly during the first month after surgery, and thereafter at monthly intervals. Anal function was evaluated by low anterior resection syndrome (LARS) score questionnaire (36) at 1 year and reviewed by two researchers. Quality of life was evaluated using the SF-36 questionnaire, administered three months following stoma reversal surgery.

### 2.5 Procedures

**Figure 1:**
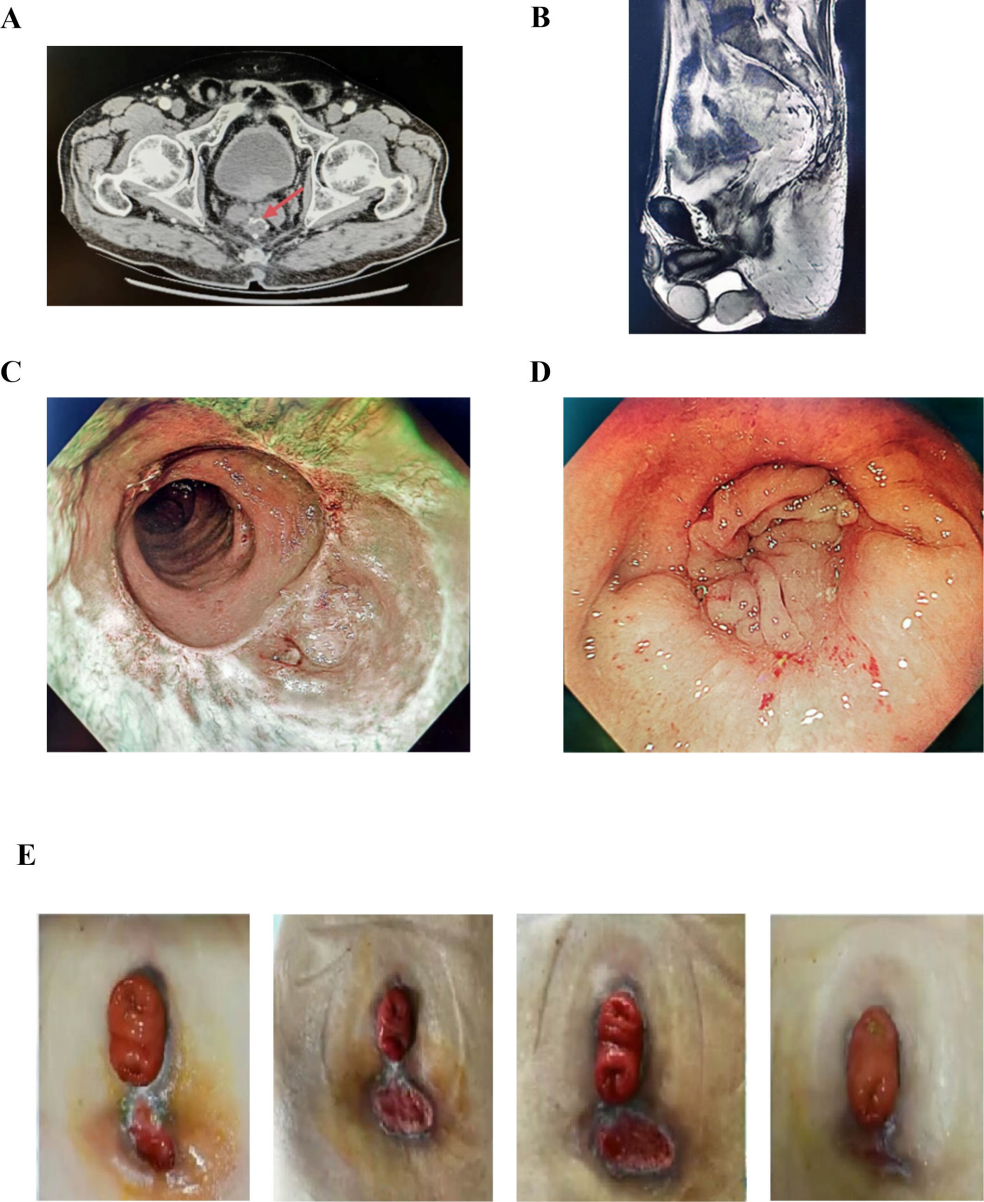
Preoperative assessment of anastomotic and stoma in patients who have undergone EC or RC. **(A–D)**. Preoperative assessment of anastomotic integrity in patients who have undergone early 3 weeks ileostomy closure. **(A)** CT demonstrates that the arrow indicates that the anastomotic site and anastomotic staples are visible, with smooth margins and no significant abnormal enhancement observed. **(B)**. MRI demonstrates that the anastomotic site has healed well, and the distal bowel is patent. **(C, D)**. electronic colonoscopy demonstrates that the mucosa at the anastomotic site is smooth, with no ulcers or masses observed; anastomotic staples are visible; the intestinal lumen is patent; **(E)**: Peristomal pyoderma gangrenosum and its treatment.

**Figure 2:**
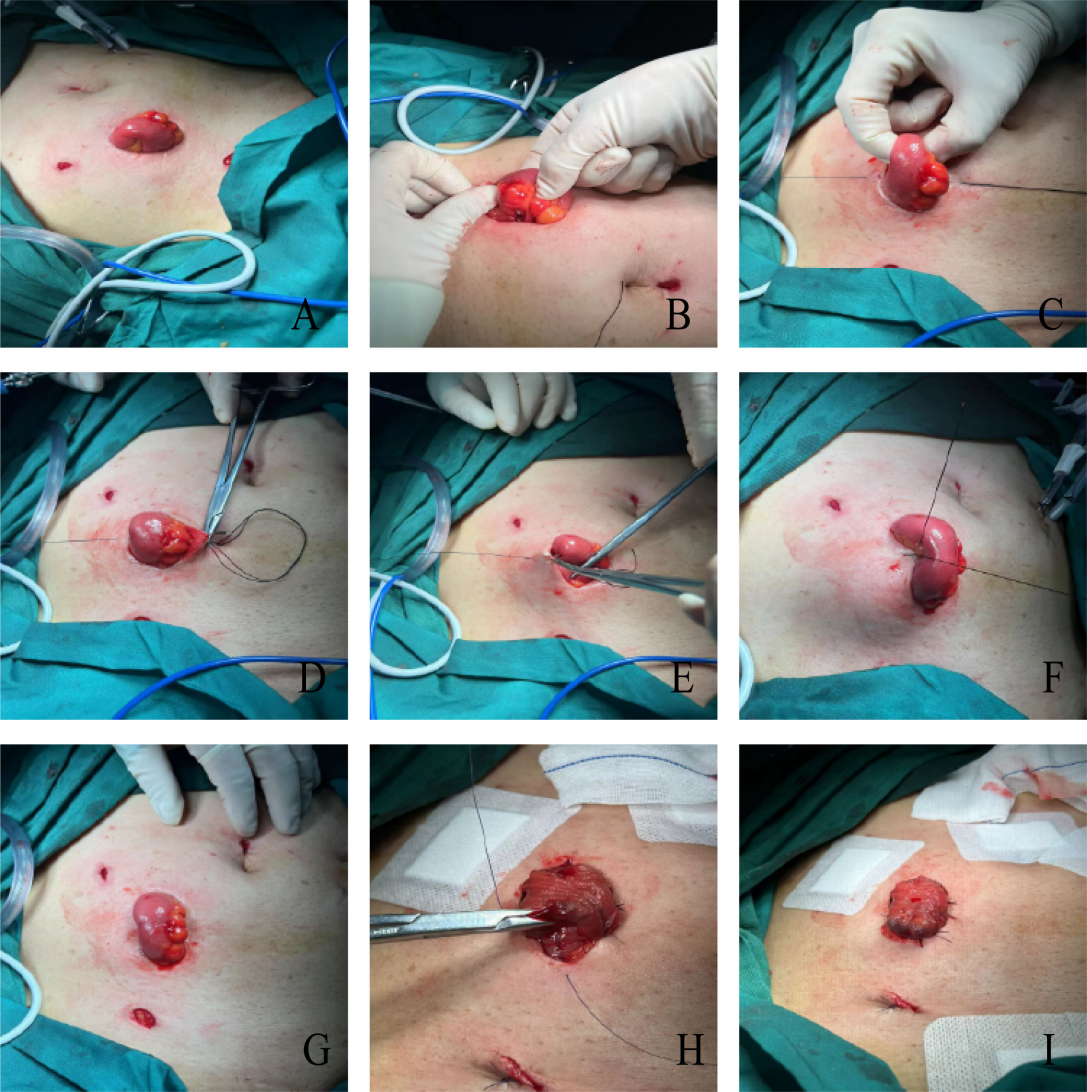
The modified one-stitch method (OM) of protective loop ileostomy. **(A)** Perform a vertical incision to advance layer by layer into the abdomen. **(B)** Propose terminal ileum. **(C)** The suture thread is passed back through the mesentery of the ileum and tied off. **(D)** Insert the needle into the skin from one side. **(E)** Puncture the skin from the opposite side. **(F)** Tighten the suture. **(G)** Make a longitudinal incision along the intestinal wall. **(H)** Secure the first stitch in place around the perimeter. **(I)** Fixed a certain number of stitches around.

All patients underwent laparoscopic radical resection of rectal cancer followed by protective loop ileostomy. For patients with upper rectal cancer, the surgical principle is partial mesorectum excision (PME), and the procedure performed was AR + PME. For those with mid or low rectal cancer, the surgical principle is total mesorectum excision (TME), and the procedure performed was LAR + TME. Some patients received NACRT, consisting of pelvic radiotherapy at a dose of 45.0-50.4 Gy, administered at 1.8-2.0 Gy per fraction over 25–28 fractions. Concurrent chemotherapy was administered during radiotherapy, with regimens including capecitabine monotherapy, XELOX or FOLFOX. Following completion of radiotherapy, patients received 1–3 cycles of consolidation chemotherapy using the same regimens. Radical surgery was performed at 8–10 weeks after NACRT. The decision to create a protective loop ileostomy was based on criteria classified into absolute and relative indications. Absolute indications, mandating routine ileostomy creation, included: (i) patient-related risk factors such as severe malnutrition, poorly controlled diabetes mellitus, prolonged corticosteroid use, or other special conditions; (ii) intraoperative uncertainty regarding anastomotic quality, defined as a positive leak test. Relative indications, requiring individualized assessment and shared decision-making between surgeon and patient, included: (i) presence of NACRT; (ii) use of more than 3 distal staple cartridges; (iii) low anastomosis (<5 cm from the anus). Patients meeting absolute indications routinely underwent diverting ileostomy, whereas those with relative indications were evaluated comprehensively with final determination made through shared decision-making. Preoperative imaging assessment confirms that stoma reversal can be performed within 2 to 4 weeks (**Figures 1A–D**). All patients underwent preoperative colonoscopy, computed tomography(CT), and MRI examinations which ruled out tumor recurrence and distant metastasis. The first postoperative assessment was performed at 2 weeks to evaluate eligibility for early closure. Patients who did not meet criteria for early closure underwent subsequent follow-up every 3 months to assess anastomotic healing. At the first follow-up visit, patients initially underwent MRI for preliminary evaluation; those with favorable MRI findings indicating good anastomotic healing then proceeded to colonoscopy, followed by CT examination. All patients underwent routine preoperative laboratory tests that included complete blood count, liver and renal function tests, coagulation profile, serum tumor markers, and a nutritional risk screen using the Nutritional Risk Screening 2002(NRS-2002) score. A score of NRS-2002 < 3 indicates no nutritional risk. Blood glucose is controlled within normal ranges. We implemented the modified one-stitch method of protective loop ileostomy in all three centers (**Figure 2**). The stoma was reversed using a traditional open abdominal technique. A fusiform incision perpendicular to the rectus abdominis muscle is made around the stoma. After mobilizing the intestine, it is transected and anastomosed using a side-to-side anastomosis with an anastomosis device. Manual reinforcement is then performed using 3–0 absorbable suture material.

The modified one-stitch method (OM) of protective loop ileostomy:a longitudinal incision is made to the right of the umbilicus through the rectus abdominis muscle, extending to the subcutaneous layer and measuring approximately 2–4 fingerbreadths in length. The anterior layer of the rectus sheath, the rectus abdominis muscle itself, and the posterior layer of the rectus sheath are incised longitudinally. The terminal ileum is then elevated through the incision, positioned approximately 30 cm distal to the ileocecal junction, with the proximal end superior and the distal end inferior. A small opening is created using an electrosurgical knife within the avascular zone of the mesentery. From the midpoint of the skin at the incision site or slightly distal to the ileum, 0.8-1.0 cm from the skin margin, insert a 1-0(United States Pharmacopeia definition) silk suture from the lateral side of the incision, exiting medially. Pass the needle and thread through the small opening in the ileal mesentery. Then, insert the needle medially at the corresponding skin site on the opposite side of the incision, exiting laterally. Following emergence near the skin margin, perform unilateral vertical mattress suturing. Pass the suture through the mesenteric orifice to the contralateral side, execute vertical mattress suturing, tighten, and knot. Retract the bowel to the midpoint or distal side, make a transverse incision, and evert the bowel for skin suturing.

### 2.6 Ethical consideration

This study was reviewed and approved by the ethics committee of The Second Hospital of Shanxi Medical University (Approval No.2025-232) and each participating institution.

### 2.7 Statistical analysis

SPSS(version 25.0) was used for statistical analyses. Continuous variables are presented as mean ± standard deviation ( $\bar{x}$ ± s) or median (interquartile range, IQR) and were compared using Student’s t test or the Mann–Whitney U test, depending on their distribution. Categorical variables are shown as n(%). Association of categorical variables was assessed using the chi-squared test or Fisher’s exact test. A Nomogram was constructed using R4.5.1 software. The model was evaluated using receiver operating characteristic curve (ROC), calibration curves, and decision curve analysis (DCA). A two-tailed *P-value* < 0.05 was considered statistically significant.

## 3 Results

### 3.1 Clinical characteristics and univariate analysis

A total of 428 consecutive patients who 94 patients in the EC group while 334 patients in RC group were identified. According to the univariate analysis, comparison of clinical characteristics revealed no statistically significant differences in Sex, BMI, Diabetes mellitus, Hypertension, (*P>0.05*), whereas characteristics including Age, pathological tumor-node-metastasis (pTNM) stage, Tumor site, and NACRT were significantly associated (*P<0.05*, **Table 1**). In the EC group, 32.9% patients (31/94) underwent AR + PME, while 67.1% patients (63/94) underwent LAR+TME. In the RC group, 24.9% patients (83/334) underwent AR combined with PME, and 75.1% patients (251/334) underwent LAR combined with TME. No statistically significant difference in the type of first surgery was observed between the two groups (χ² = 2.480, P = 0.115).

**Table 1:** Clinical characteristics and univariate analysis of study subjects before PSM.

Variable	Total (n = 428)	EC (n = 94)	RC (n = 334)	Statistic	*P*	SMD
Age	64.50 ± 13.69	58.74 ± 15.22	66.11 ± 12.79	-4.288	0.001	0.576
BMI	23.13 ± 3.05	23.14 ± 2.93	23.13 ± 3.08	0.045	0.964	-0.005
Sex, n (%)				1.254	0.263	
female	167 (39.0)	32 (34.0)	135 (40.4)			0.130
male	261 (61.0)	62 (66.0)	199 (59.6)			-0.130
pTNM Stage, n (%)				11.889	0.003	
I	80 (18.7)	28 (29.8)	52 (15.6)			-0.392
II	155 (36.2)	35 (37.2)	120 (35.9)			-0.027
III	193 (45.1)	31 (33.0)	162 (48.5)			0.311
Tumor site, n (%)				7.460	0.024	
Lower	114 (26.6)	15 (16.0)	99 (29.6)			0.300
Middle	200 (46.8)	48 (51.0)	152 (45.5)			-0.112
Upper	114 (26.6)	31 (33.0)	83 (24.9)			-0.188
NACRT, n (%)				17.681	0.001	
No	309 (72.2)	84 (89.4)	225 (67.4)			-0.469
Yes	119 (27.8)	10 (10.6)	109 (32.6)			0.469
Hypertension, n (%)				0.220	0.639	
No	292 (68.2)	66 (70.2)	226 (67.7)			-0.054
Yes	136 (31.8)	28 (29.8)	108 (32.3)			0.054
Diabetes mellitus, n (%)				1.003	0.317	
No	359 (83.9)	82 (87.2)	277 (82.9)			-0.114
Yes	69 (16.1)	12 (12.8)	57 (17.1)			0.114

### 3.2 Construction and validation of nomogram

After incorporating statistically significant factors from the univariate analysis into the multivariate logistic analysis, the results indicated that age, pTNM stage, Tumor site, and NACRT were independent influencing factors for EIC, as shown in **Table 2**. Specifically, Stage I (OR = 2.569, 95%CI: 1.334–4.945, *P=0.005*) had significant protective effects on EIC compared to Stage II and III(P<0.05). Lower rectal cancer (OR = 0.347, 95%CI: 0.168–0.718, *P=0.004*) had a significant impact on EIC compared to those with Middle/Upper cancer, serving as an independent risk factor. Additionally, receiving NACRT (OR = 4.490, 95%CI: 2.157–9.347, *P=0.001*) had a significant effect on EIC compared to those with not receiving NACRT, serving as an independent risk factor (**Table 3**). The calculation results of collinearity analysis indicated no collinearity.

**Table 2:** Clinical characteristics after PSM.

Variable	Total (n = 178)	EC (n = 89)	RC (n = 89)	Statistic	*P*	SMD
Age	60.72 ± 14.30	59.87 ± 14.61	61.58 ± 14.02	0.801	0.424	0.123
BMI	23.07 ± 3.05	23.21 ± 2.98	22.93 ± 3.13	0.615	0.539	-0.090
Sex, n (%)				0.606	0.436	
female	65 (36.5)	30 (33.7)	35 (39.3)			0.115
male	113 (63.5)	59 (66.3)	54 (60.7)			-0.115
pTNM, n (%)				0.445	0.800	
I	50 (28.1)	27 (30.3)	23 (25.8)			-0.103
II	66 (37.1)	32 (36.0)	34 (38.2)			0.046
III	62 (34.8)	30 (33.7)	32 (36.0)			0.047
Tumor site, n (%)				1.204	0.548	
Lower	35 (19.7)	15 (16.8)	20 (22.5)			0.135
Middle	92 (51.7)	46 (51.7)	46 (51.7)			0.000
Upper	51 (28.6)	28 (31.5)	23 (25.8)			-0.128
NACRT, n (%)				0.771	0.380	
No	154 (86.5)	79 (88.8)	75 (84.2)			-0.123
Yes	24 (13.5)	10 (11.2)	14 (15.8)			0.123
Hypertension, n (%)				0.026	0.872	
No	121 (68.0)	61 (68.5)	60 (67.4)			-0.024
Yes	57 (32.0)	28 (31.5)	29 (32.6)			0.024
Diabetes mellitus, n (%)				0.393	0.531	
No	151 (84.8)	77 (86.5)	74 (83.1)			-0.090
Yes	27 (15.2)	12 (13.5)	15 (16.9)			0.090

**Table 3:** Multivariate logistic regression analysis of factors associated with EIC.

Variables	SE	Wald	*P* value	OR	95%CI
Age	0.010	21.868	0.001	0.956	0.938-0.974
Tumor site					
Lower	0.370	8.156	0.004	0.347	0.168-0.718
Middle	0.296	0.966	0.326	0.748	0.419-1.335
Upper*					
pTNM stage					
I	0.334	7.969	0.005	2.569	1.334-4.945
II	0.296	1.222	0.269	1.387	0.777-2.477
III*					
NACRT					
Yes	0.374	16.119	0.001	4.490	2.157-9.347
No*					

*Is the reference category.

**Figure 3:**
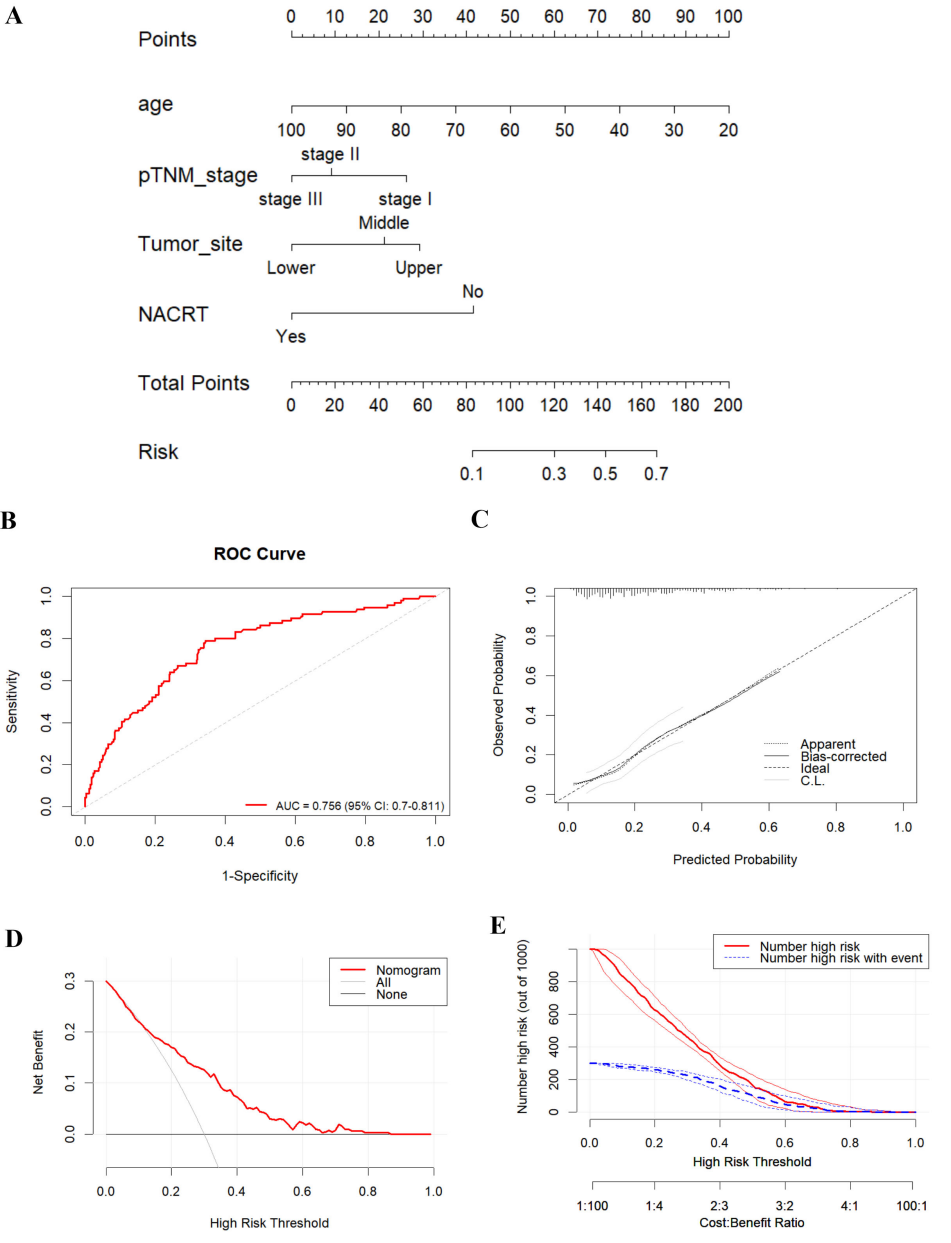
Construction and validation of nomogram for predicting EIC. **(A)** Nomogram prediction model for EIC. **(B)** ROC of the Nomogram prediction model. **(C)** Calibration curve of the Nomogram prediction model. **(D)** The net benefit curve of the nomogram. **(E)** DCA for the Nomogram prediction model.

Based on the statistically significant risk factors identified through logistic regression analysis, a Nomogram prediction model (**Figure 3A**) was constructed. This nomogram targets the early closure of ileostomy, incorporating four core indicators: age, pTNM stage, tumor location, and NACRT. Based on the patient’s actual clinical data, the corresponding scores for each of the four indicators are retrieved and summed to obtain a total score. This total score is then mapped to the “Risk Probability of Early Closure” axis to get the specific probability value. Finally, the quantitative risk is determined by combining the risk threshold set in the study. A higher total score indicates a higher risk probability of EC, while a lower total score corresponds to a lower risk probability of EC.

### 3.3 Evaluation of model-based risk stratification and its clinical applicability

The results of ROC curve (**Figure 3B**) show that the Area Under the Curve area under the curve (AUC) of the model is 0.756, with a corresponding 95% confidence interval of 0.70-0.811, indicating that the model has a moderate-to-high discriminative efficacy in distinguishing between “early closure” and “routine closure”. The calibration curve (**Figure 3C**) of the predictive model for ileostomy closure timing after rectal cancer surgery assesses the consistency between the model’s predicted probabilities and the actual occurrence probabilities. The high degree of overlap among the “Apparent” curve, “Bias-corrected” curve, and “Ideal” curve in the figure indicates that the model’s predicted probabilities are well-matched with the actual occurrence probabilities of “early closure”. The DCA (**Figure 3D**) results showed that the net benefit of the model is significantly higher than that of the strategies of “classifying all cases as early closure” and “classifying all cases as routine closure”.This indicates that using this nomogram to predict closure timing can bring greater net benefits to clinical decision-making. The risk stratification and cost-benefit analysis (**Figure 3E**) show that it illustrates trends through a solid red line (total number of patients classified as high-risk) and a dashed blue line (number of true positives classified as high-risk who actually underwent early closure). As the threshold increases, both curves decrease and the gap between them narrows, indicating a reduction in the size of the high-risk population alongside an improvement in judgment accuracy. Overall, this helps clinicians balance “coverage of identifying patients suitable for early closure” and “accuracy of reducing misclassification”.

### 3.4 Primary and secondary outcome

**Figure 4:**
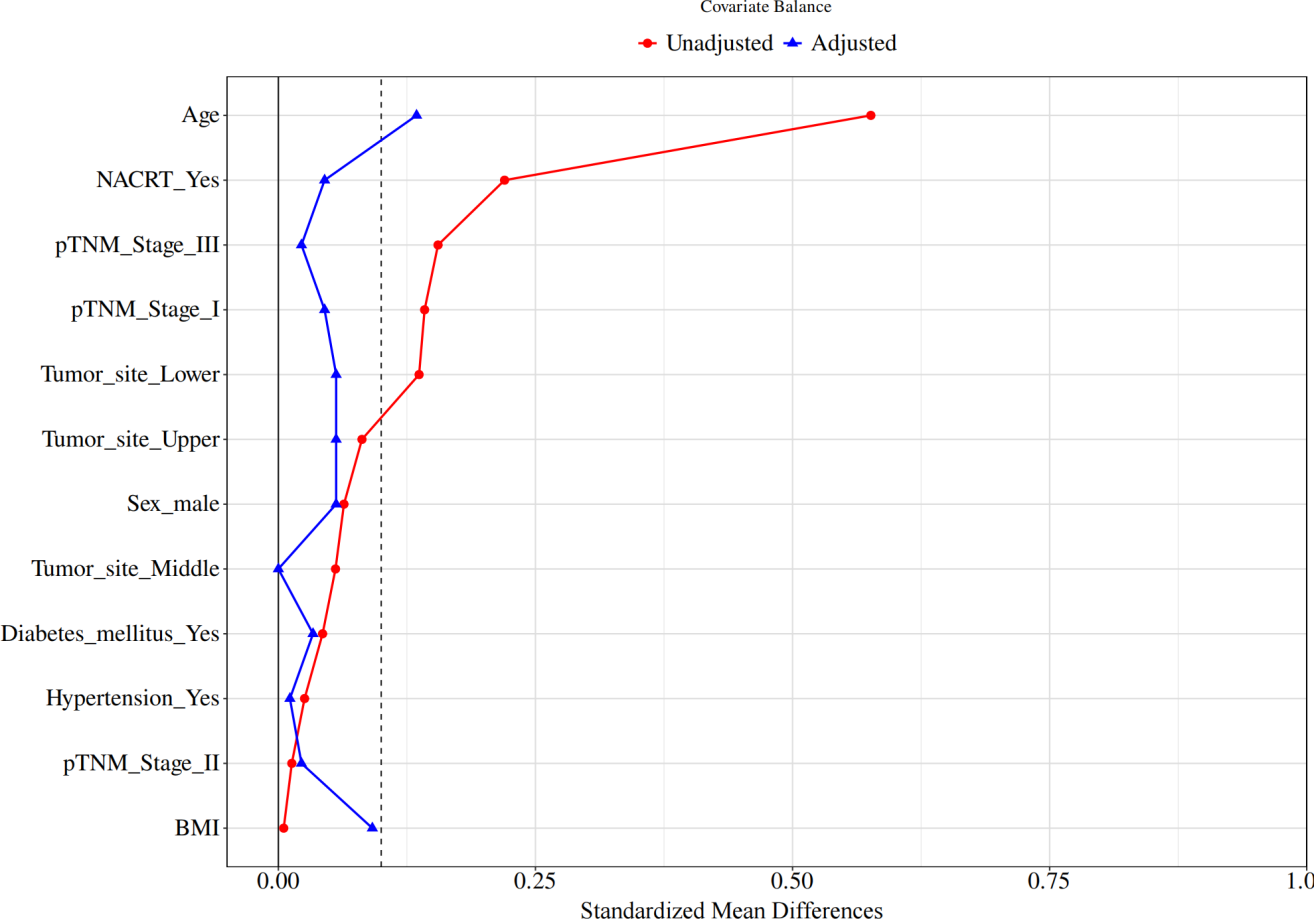
Standardized mean difference of variables before and after PSM. PSM, propensity score matching; SMD, standardized mean difference; NACRT, neoadjuvant chemoradiotherapy; pTNM, pathological tumor-node-metastasis.

In our study, the PSM method with a caliper value of 0.25 was applied to reduce the influence of confounding variables between the EC and RC groups.89 pairs of rectal patients were matched. There were no statistical differences in the baseline characteristics between the two groups after PSM (**Table 2**; **Figure 4**). There was no statistically significant difference in the incidence of post-operative complications and LARS scores between both groups (*P>0.05*). The EC group exhibited a lower incidence of stoma-related complications and shorter operative time (*P<0.05*) compared to the RC group. SF-36 quality of life score in EC group was significantly higher (*P<0.001*) than those in the RC group (**Table 4**). Notably, one rare case of peristomal pyoderma gangrenosum occurred in the RC group (**Figure 1E**). In the EC group, 10.11% of patients(n=9) delayed the adjuvant chemotherapy due to complications, including postoperative incision infection, anastomotic leakage, and temporary intestinal obstruction compared to 13.48% (n = 12) in the RC group. There was no statistically significant difference in the impact on adjuvant chemotherapy between the two group (χ² = 0.225, P = 0.635).

**Table 4:** Primary and secondary outcome of study subjects.

Variables	EC(n = 89)	RC(n = 89)	Statistic	*P*
Post-operative complications	27(30.3)	28(31.5)	0.260	0.871
postoperative incision infection	5(5.6)	3 (3.4)	0.131	0.718
electrolyte disorder	18(20.2)	16(18.0)	0.145	0.703
anastomotic leakage	0	1(1.1)	-	-
temporary intestinal obstruction	4(4.5)	8(9.0)	1.430	0.232
Stoma-related complications	11(12.3)	31(34.8)	12.465	0.001
stoma retraction	4(4.5)	6(6.7)	0.424	0.515
parastomal hernia	1(1.1)	9(10.1)	6.781	0.009
stoma prolapse	2(2.2)	3 (3.4)	0.004	0.948
fecal dermatitis	1(1.1)	2(2.2)	–	–
rectal anastomotic stricture	3(3.4)	10(11.2)	4.066	0.044
pyoderma gangrenosum	0	1(1.1)	–	–
LARS	29(21.5,34)	28(22.5,33)	0.288	0.773
Operative time	86 (78,95)	95(80.5,115.5)	2.996	0.003
Time until first exhaust	2.46 ± 0.62	3.23 ± 0.75	0.791	0.443
Time until first defecation	3.78 ± 0.65	4.18 ± 1.08	0.751	0.371
Total length of hospital stays	12.62 ± 2.26	14.28 ± 3.24	0.420	0.675
SF-36 quality of life score	112.16 ± 8.42	90.56 ± 4.92	2.215	0.028

## 4 Discussion

We conducted a retrospective, multicenter study to compare the clinical efficacy and safety of early closure versus routine closure and influencing factors. In this study, our results showed that early closure (2–4 weeks) of ileostomy after modified ostomy technique in patients with rectal adenocarcinoma is a safe and feasible technique which is associated with favorable outcome.

In this study, the overall postoperative complication rate did not increase in the EC group, indicating that EIC (2–4 weeks) is safe and feasible. The incidence of stoma-related complications was significantly lower than in the RC group (12.3% vs. 34.8%, P<0.05), suggesting that prolonged stoma duration increases susceptibility to complications due to persistent irritation from excretions.Both groups underwent modified stoma techniques, reducing the incidence of stoma dermatitis and stoma prolapse. After PSM, the EC group and RC group showed comparable times to first bowel movement and flatus, as well as similar total length of hospital stays. This indicates that intestinal functional recovery is largely equivalent between the two closure strategies in appropriately selected patients. Stoma reversal is the most effective treatment to restore intestinal continuity. EIC more rapidly restored abdominal wall continuity and integrity, potentially explaining the reduced incidence of parastomal hernia. Patients in RC group experienced longer stoma intervals, during which the anastomotic site, deprived of mechanical distension from faecal passage, was prone to atrophy and scar contracture. Adjuvant chemotherapy is associated with a higher risk of post-operative complications, particularly with wound complications (37). Therefore, both groups exhibited a relatively high rate of postoperative wound infection. The decreased incidence of stoma prolapse using modified approach is attributed to the suturing of the anterior rectus sheath at both ends of the incision, which provides additional fascial support and prevents telescoping of the bowel (34). Furthermore, the elevated stoma position relative to the abdominal wall surface minimizes skin contact with effluent, reducing the risk of peristomal dermatitis (35). Some studies have suggested that ileostomy may not reduce the incidence of AL (4), and this remains controversial. However, the fecal diversion effect significantly mitigates the severity of complications resulting from AL, such as abdominal-pelvic infection and abscess formation, and decreases the need for unplanned secondary surgery (38, 39). Although the incidence of AL is very low in upper rectal cancer, we performed ileostomy in patients with upper rectal tumors based on intraoperative findings (positive leak test) or significant comorbidities, prioritizing safety considerations. Patients in the RC group with anastomotic stenosis had undergone NACRT, which may have contributed to fibrosis and stricture formation. The stenosis in these patients may have been caused by an unrecognized or clinically silent anastomotic leak, particularly given the context of neoadjuvant radiotherapy and low anastomoses. The primary treatment was balloon dilation as the initial intervention. Re-operation was performed only when balloon dilation proved ineffective. The stenoses primarily involved low anastomoses, consistent with the higher risk profile of these patients. The multicenter study was conducted within a single region (Taiyuan, Shanxi Province, China), minimizing variation in surgical expertise across centers.

The time of preventive ileostomy closure should not be fixed at a specific point in time. There are conflicting results rooted in patients’ heterogeneity. It is more reasonable to determine the timing of stoma reversal based on patient subgroups. Nevertheless, we have proposed a promising strategy called “stratified closure” to guide patient selection and sequencing of treatment. The Nomogram prediction model that incorporates age, tumor site, NACRT and pTNM stage can effectively predict EIC in rectal cancer.

In our study, multivariate analysis revealed that age, tumor site, NACRT, and pTNM stage are independent factors influencing EIC. Middle-aged and young patients, mid-to-high rectal tumors, absence of NACRT, and stage I rectal tumors are associated with a tendency toward early closure. Thus, patient selection combining age, tumor site, NACRT, and TNM stage can improve the decision-making process. Recent research has reported that patients with signs of AL, diabetes mellitus and steroid treatment are not suitable for EIC (40). However, in our study, diabetes did not show statistical significance in either the EC or RC groups. This may be attributed to the fact that diabetic patients can undergo EC provided their blood glucose levels are well controlled through medication or insulin. With improved surgical techniques and meticulous care, EIC(2-4weeks) is safe and feasible, which is supported by previously published protocols. A meta-analysis indicates EC had higher odds of AL than RC when early closure was done ≤2 weeks (OR: 2.12, P = 0.047) but not within 3 to 4 weeks (OR: 2.98, P = 0.107) (23). Malik et al. (41) found that approximately 63% of patients with a protective ileostomy develop stoma-related complications in the early postoperative period (within 30 days). Stoma-related complications require frequent medical visits and treatment (42). Among these patients, young and middle-aged individuals have a lower acceptance of stoma life compared to the elderly. The EC group generally had a younger age than RC group, which also reflects the urgent need of them to shorten the duration of stoma life. Low anastomoses have a high incidence of LARS. Except for cases where patients cannot tolerate stoma life, EIC is generally not recommended. This is to avoid adverse effects of anal incontinence, diarrhea, or constipation on the healing of the anastomosis. It is generally believed that most AL occur 5–7 days after surgery, and the risk of AL gradually decreases as the recovery period extends (43). However, patients who undergo EIC still face the risk of delayed AL. To improve surgical safety, we have implemented a series of measures to reduce the incidence of AL (27, 29–31).

There is significant controversy regarding the timing of stoma reversal in patients undergoing NCART. Some studies reported that time to stoma closure was nearly doubled when patients underwent NACRT (44). It significantly affect adversely the healing of anastomotic sites, prolonged the time required for closure and increased the probability of permanent stoma (45). Preoperative radiotherapy increased incidence of stenosis or stiffness proximal to anastomosis in rectal cancer patients with radical resection and diverting ileostomy, which can account for the 3–6 months closure. On one hand, it is argued that after NACRT, local tissue edema, microcirculatory disorders, and impaired tissue regeneration capacity may occur (46). These conditions can lead to complications such as radiation enteritis postoperatively, resulting in delayed healing or non-healing of the anastomosis (47). Secondly, EIC is prone to complications like incisional infection, which may cause interruption or delay of postoperative chemoradiotherapy. To manage postoperative and stoma-related complications, stoma reversal has to be postponed. In contrast, RC can ensure the safety of the anastomosis. On the other hand, some study suggested that there was no significant correlation between rectal AL and NACRT (48). Treatment delays caused by stoma closure during chemotherapy do not affect treatment efficacy. Furthermore, the TNM stage is closely associated with whether a patient receives NACRT (49). Patients with Stage I typically do not require postoperative chemotherapy, recover more quickly, and can meet the criteria for stoma reversal earlier. In contrast, patients with Stage II-III need adjuvant chemotherapy postoperatively to prevent recurrence and metastasis. EIC in these patients may disrupt the completeness of adjuvant chemotherapy. Therefore, to ensure favorable oncological outcomes and higher survival rates, RC is preferred. In clinical practice, considering anastomotic safety of chemoradiotherapy, most clinicians still advocate for delayed stoma reversal. We argue that patients receiving NACRT should be taken into account. When the anastomosis is assessed to be well-healed, EIC should be performed as early as possible. This is to avoid severe rectal anastomotic stricture. However, for patients at high risk of postoperative radiation enteritis and metastatic recurrence, RC represents a safer option.

Ileostomy not only increases psychological and financial burdens on patients but also significantly raises medical risks and societal costs. The necessity of stoma is a significant concern. Recent studies have identified some novel surgical techniques to avoid stoma. The stent-based diverting technique (SDT) is a novel biodegradable device to avoid a diverting stoma (50). In a prospective study of 34 patients treated with LAR followed by SDT, it fully degraded within 21 (18–24) days and only 1 patient had anastomotic leakage (6). Bugiantella et al. (51) carried out a temporary percutaneous ileostomy by a jejunal probe introduced in the distal ileum, that can be removed without a surgical procedure and with negligible complications. The 2-stage pull-through hand-sewn coloanal anastomosis could be considered as a surgical alternative (52, 53). Bianco et al. (54) developed a “Short stump and High anastomosis Pull-through” procedure for delayed coloanal anastomosis without a stoma. CG-100 that can be decomposed naturally *in vivo* provides a safe method for fecal diversion over a newly created anastomosis (55). Adopting different techniques based on the specific condition of the patient can lead to greater benefits.

Our study demonstrated that early closure does not increase postoperative complications and reduces stoma-related complications, which is in accordance with the research reported by Park et al. (56) and Ellebæk (57) et al. Early closure within 2–4 weeks is currently less studied. Additionally, our results supported the safety and feasibility of early closure(2–4 week window) in meticulously selected patients with intact anastomoses and uneventful postoperative courses, supporting the conclusions of Bhullar (58) et al. Furthermore, we developed a nomogram prediction model to identify specific patient phenotypes most likely to benefit from early closure; we improved the operative technique for early closure; we performed PSM to minimize selection bias and confounding.

The 2–4 week window offers advantages over established 2-week or 4-week protocols. A mixed-methods, cross-sectional study of North American patients and surgeons indicated that patients regarded two operations in two weeks being too taxing on the body was deemed the biggest disadvantage (24). Although 72.9% (35/48) of patients expressed willingness for closure within 2 weeks, 27.1% (13/48) considered this interval unacceptable (24). In contrast, the 2–4 week interval provides a more extended recovery period between the index resection and stoma reversal, thereby improving patient acceptability and reducing the psychological and physical burden associated with closely spaced major abdominal procedures. Compared to closure within 2 weeks, the 2–4 week window affords patients a more prolonged period of nutritional support, enabling the correction of anemia and restoration of serum albumin to normal or near-normal levels. Compared to the 4-week protocol, the 2–4 week closure window enables stoma reversal prior to the initiation of adjuvant chemotherapy, which may benefit from long-term oncological survival.

The study has some limitations. This retrospective study was not randomized, which may introduce potential bias; Additionally, the study may have limited generalizability to patients with very low BMI (~23). We will next conduct a multicenter randomized controlled trial (RCT) of early closure. Future research will investigate the applicability of early closure in specific patient populations, including those with low BMI. The sample size for EIC remains limited, and future studies will expand this cohort for further investigation. Moreover, Ileostomy is not necessary for most patients who undergo colorectal anastomosis, but rather is a protective option for patients with a high risk of anastomotic leakage (59, 60).We should reduce unnecessary ileostomy or explore alternative stoma management approaches. Where ostomies are performed, every effort should be made to offer early closure to eligible patients. Early ileostomy closure can only be performed in carefully selected patients and should not be undertaken routinely (11). Early closure of ileostomy at 2–4 weeks may be a more effective strategy. We propose that stratified closure is the trend of the future, with the potential to become a prevailing approach in future practice.

## 5 Conclusion

Early closure of ileostomy at 2–4 weeks is safe and beneficial in selected patients. It does not increase the incidence of postoperative complications and reduces the rates of parastomal hernia and rectal anastomotic stricture. The Nomogram prediction model that incorporates age, tumor site, NACRT and pTNM stage can effectively predict early closure of ileostomy at 2–4 weeks in rectal cancer, and can perform effective preoperative stratification to guide clinical decision-making.

## Data Availability

The raw data supporting the conclusions of this article will be made available by the authors, without undue reservation.
